# Case Report: A novel *HNRNPK* gene variant causing Au-Kline syndrome; the genotype and phenotype of the oldest individual ever described

**DOI:** 10.3389/fpsyt.2026.1864501

**Published:** 2026-08-07

**Authors:** Anja G. Bos-Roubos, Rosalie te Brinke, Anja A. Kattentidt-Mouravieva, Rolph Pfundt, Laura C.G. de Graaff, Ellen Wingbermühle, Jos I.M. Egger

**Affiliations:** 1 Center of Excellence for Neuropsychiatry, Vincent van Gogh Institute for Psychiatry, Venray, Netherlands; 2 Donders Institute for Brain, Cognition, and Behaviour, Radboud University, Nijmegen, Netherlands; 3 Stichting Zuidwester, Middelharnis, Netherlands; 4 Human Genetics, Radboud University Medical Center, Nijmegen, Netherlands; 5 Center for Adults with Rare Genetic Syndromes, Department of Internal Medicine, Erasmus University Medical Center, Rotterdam, Netherlands; 6 Center for Adults with Rare Genetic Syndromes, Department of Internal Medicine, Radboud University Medical Center, Nijmegen, Netherlands; 7 Stevig Specialized and Forensic Care for People with Intellectual Disabilities, Dichterbij, Oostrum, Netherlands

**Keywords:** Au-Kline syndrome, case report, contextual neuropsychology, genetic testing, psychomotor interventions

## Abstract

This report describes the diagnostic and treatment course of an adult woman with the ultra-rare Au-Kline syndrome (AUKS). Since the age of 32 years, when the syndrome was yet undiagnosed, the patient suffered from behavioral decline coinciding with changes in living conditions. At the age of 38, following a bladder infection, she presented with a delirium for which antipsychotic medication was started. The delirium resolved but, while the medication was continued, the pre-existing behavioral symptoms persisted. Clinicpal genetic evaluation revealed a history of congenital hypotonia, developmental delay, and multiple (craniofacial) dysmorphisms. Subsequent trio-based genetic testing demonstrated a novel pathogenic variant in the *HNRNPK* gene, causative for AUKS. Clinical neuropsychological assessment showed moderate intellectual disability, slow information processing speed, restricted verbal expression, and limited comprehension. Registration of sensory information was severely hampered and there was difficulty in self-modulating the sensory information to be processed. Contextual adaptations and individual psychomotor therapy were then implemented to optimize sensory and motor information processing, to promote daily functioning, and reduce behavioral decline. These interventions, along with the long-term psychotropic medication, had beneficial effects on her functioning, which subsequently returned to near premorbid levels by the age of 45. Overall, this case underscores that the genetic and neuropsychological identification of a neurodevelopmental disorder, together with the integration of tailored psychomotor interventions and contextual adaptations, can improve psychiatric management and daily functioning, and mitigate behavioral decline in adulthood, even following a prolonged diagnostic delay.

## 1 Introduction

Au-Kline syndrome is an ultrarare syndrome of which only 75 individuals have been reported worldwide (1). The oldest individual published as yet was 32 years old (2).

Au-Kline syndrome (AUKS; OMIM #616580), also known as Okamoto syndrome (2–4), was first described by Au et al. in 2015 and is caused by heterozygous loss-of-function variants in the *HNRNPK* gene on chromosome 9q21 that encodes the heterogeneous nuclear ribonucleoprotein K (OMIM #600712). AUKS is characterized by hypotonia, global developmental delay, characteristic facies (long palpebral fissures, shallow orbits, ptosis, a broad nasal bridge, hypoplastic alae nasi, downturned corners of the mouth, and a long face), congenital heart defects, genitourinary abnormalities, skeletal abnormalities, and variable other congenital malformations (2). In several cases, though not in all, specific brain abnormalities (e.g., hypomyelination, (partial) corpus collosum agenesis, asymmetric lateral ventricles) have been reported (5).

Of the 75 patients with *HNRNPK* variants ever reported, 24 individuals (age range 12 days - 17 years) have been described in terms of their physical/medical characteristics and clinically observed developmental problems. A literature overview of the characteristics of these 24 individuals is presented in **Supplementary File 3 (part a)**. Here, we describe the genotype and phenotype (including cognition, behavior, and psychopathology) of AUKS in an adult female based on both genetic testing and formal clinical neuropsychological assessment.

## 2 Case description

### 2.1 Patient presentation

At the time of the presentation, the patient was a 38 years old woman referred by her general practitioner (GP) to the physician of an outpatient clinic for intellectual disability (ID) because of behavioral decline.

The patient was born at 41 weeks of gestation as the first child of a Dutch father and a South American mother. Family history was unremarkable, with both parents in good health and one unaffected brother. Her parents reported a postnatal cerebral hemorrhage, and hypotonia without feeding or drinking difficulties. During the first two years of her life, to improve her delayed motor function, she underwent physiotherapy. Brief episodes of arms and legs stretching together with upward gazing had been observed since childhood. The patient had never been diagnosed with epilepsy and was generally healthy.

Intellectual disability was diagnosed at the age of two. Speech had always been nonfluent and agrammatical. The patient received special education until the age of 20. Nonverbal intelligence tests at the age of 29 years revealed developmental age equivalents of 46–61 months. At the age of 38 years, developmental age was lower than 31–84 months.

Up to the age of 32 years, she lived with her parents and attended a day care facility. Both at home and within the day care setting, she was able to perform simple, structured household tasks under supervision, such as setting the table, loading the dishwasher, and cutting vegetables. She was also involved, under supervision, in training other clients to perform these same activities. Her parents described her as a loving and beloved person, with a mild temperament.

**Figure 1:**
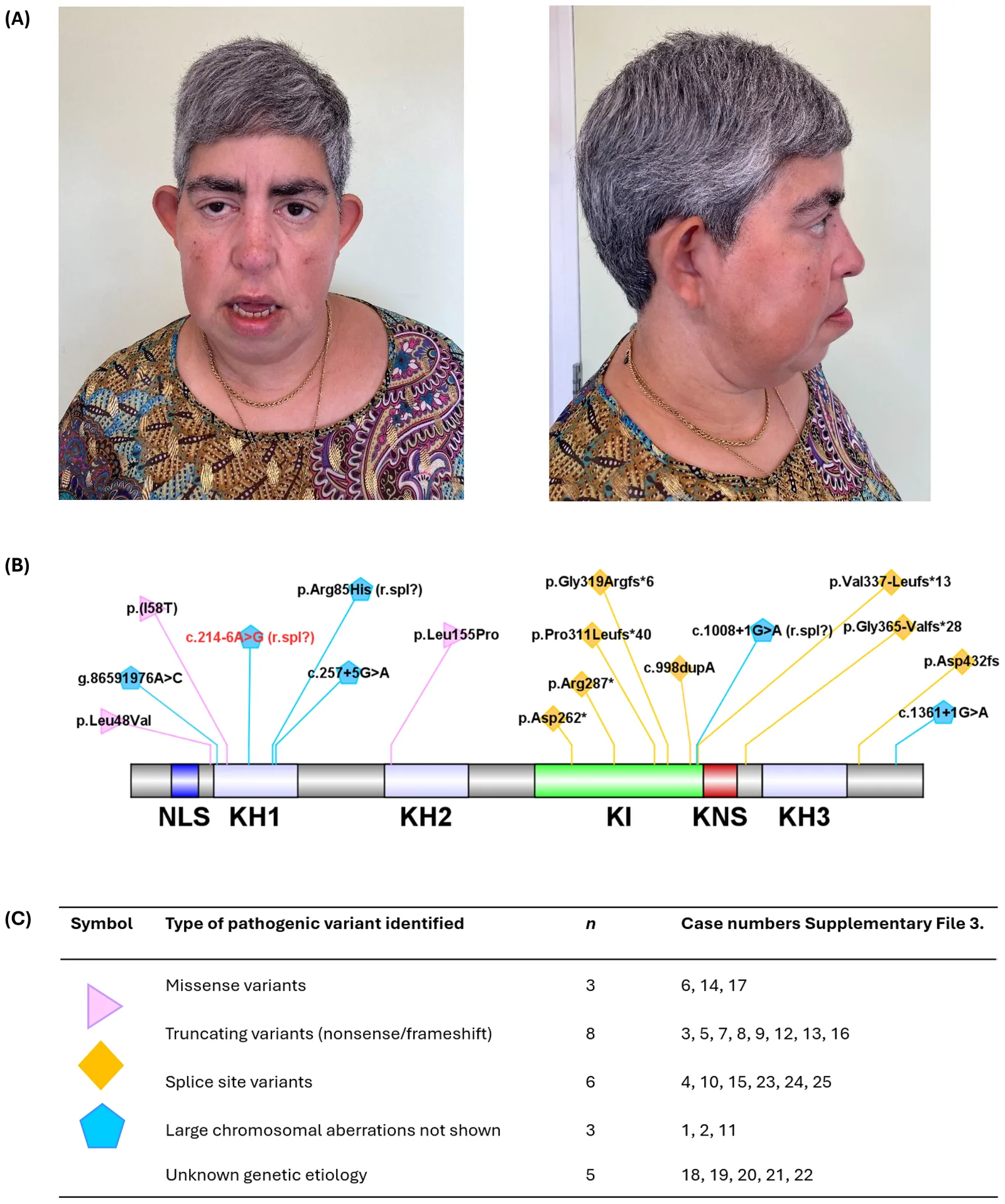
**(A)** Consent for publication of the photographs was provided. The patient at the age of 45 years. Characteristic facies: hypotonia, low posterior hairline, long face, long palpebral fissures, prominent ears, irregular teeth, and prognathism. **(B)** Structure of heterogeneous nuclear ribonucleoprotein, depicting three K homology (KH) domains, one N-terminal bipartite nuclear localization signal (NLS), one K-protein interactive region (KI) domain, and one nuclear – cytoplasmic shuttling domain (KNS). The patient in red text. **(C)** Summary.

On physical examination at 38 years of age, the patient presented with short stature (-2.5SD), normal head circumference (+0.4SD), hypertrichosis of the scalp, and hypoplasia of the fifth fingernails. Hypotonia was still present. She presented with multiple craniofacial dysmorphisms, as shown in **Figure 1**. The patient’s characteristics are summarized in **Supplementary File 3 (part b)**.

### 2.2 Clinical course

At the age of 32, shortly after moving from her parents’ home to an assisted living facility, the patient’s behavior deteriorated. Symptoms included withdrawn behavior, disorientation, restlessness and pacing, apathy, gesticulating with arms and legs, inability to communicate with caregivers, talking aloud to herself, confabulation, disrupted day/night cycle, loss of hygiene skills, and regression in activities of daily living. During subsequent years, her symptoms gradually worsened. Hypothyroidism, anemia, electrolyte disturbances, hepatic or renal dysfunction, and epilepsy were ruled out. As she did not use any medication, no side effects were expected.

At the age of 35, the patient returned to live with her parents, but this did not reverse the symptoms. A psychiatrist hypothesized ‘detachment trauma’, post-traumatic stress syndrome, autism, and dementia. A neurological examination at age 37, including brain MRI scan, ruled out clinically significant abnormalities. There were no radiologic signs of dementia.

At the age of 38, she developed a delirium during a bladder infection for which the GP started the antipsychotic drug pipamperone. In the same year, regression due to genetic conditions was postulated by the ID physician. At the age of 40, the dose of pipamperone was increased (66 mg/day), because of persistent motor symptoms (gesticulating with the arms, drawing with the thumb and index finger on her own legs) and repetitive self-talk (apparently talking to someone “in her head”).

## 3 Table-time line

**Figure 2:**
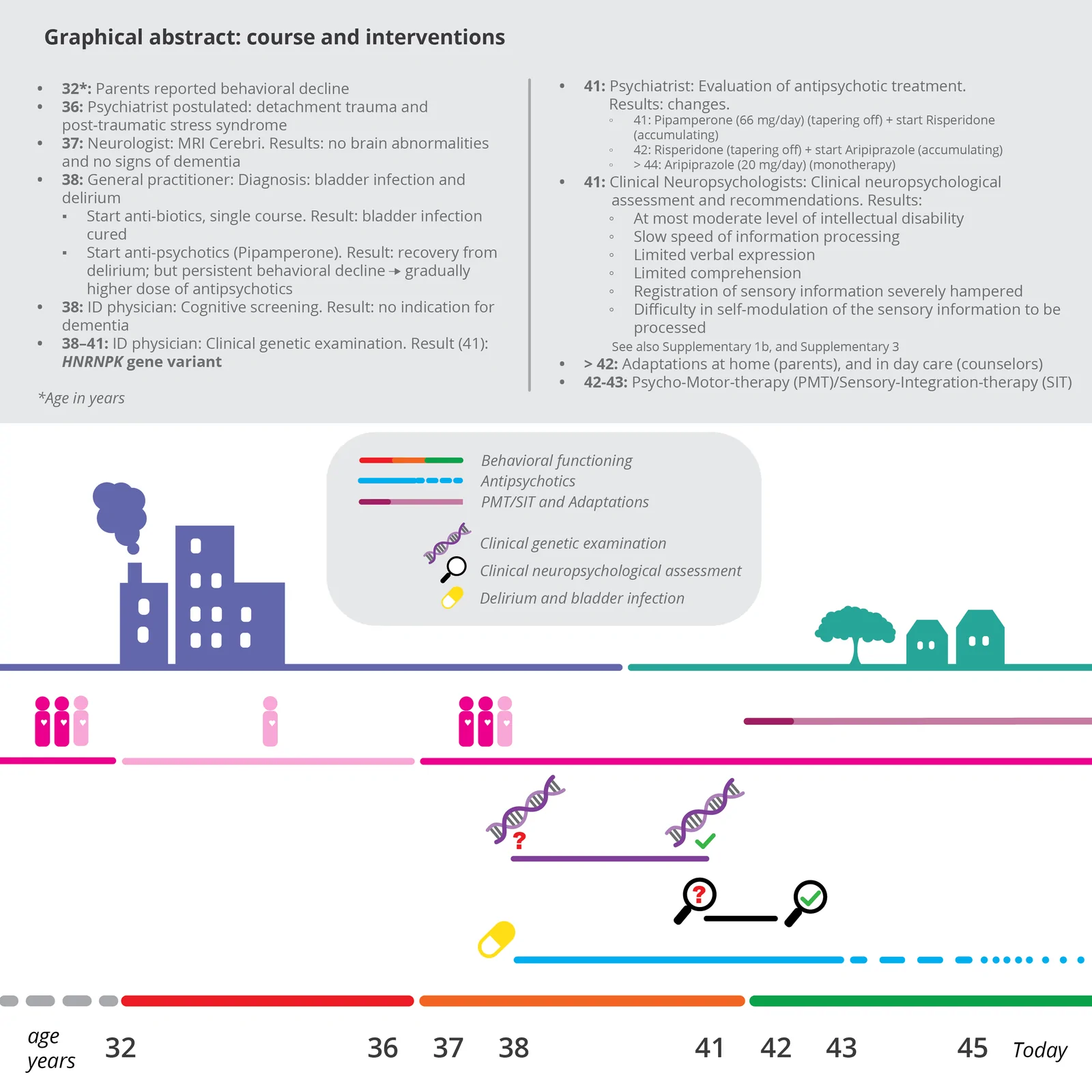
Graphical abstract of the patient's clinical course and the interventions applied.

**Figure 2** provides a graphical abstract of the patient’s clinical course and the interventions applied.

## 4 Diagnostic assessment and therapeutic interventions

### 4.1 Materials and methods

The patient’s legal representatives provided written informed consent prior to the following interventions. We performed trio-based whole-exome sequencing (WES), as described in **Supplementary File 1**. The differential diagnosis included Kabuki syndrome, Coffin–Siris syndrome, and an inborn error of metabolism.

Clinical neuropsychological assessment was performed, using the instruments as described in **Supplementary File 2**. As part of the assessment, we performed a detailed heteroanamnesis regarding her developmental history and personal characteristics. In addition, we performed a systematic analysis of video recordings of the patient’s behavior at home.

### 4.2 Genetic testing

At the age of 41, whole-exome sequencing revealed a *de novo* heterozygous intronic variant in the splice acceptor region of intron 5 of the *HNRNPK* gene, NM_002140.5; c.214-6A>G (r.spl)?. This variant has not been previously described in the literature or in variant databases such as gnomAD V4.1. Splice prediction tools predicted a possible negative effect of this variant on the splice acceptor site. Additional RNA isolation from blood lymphocytes and subsequent cDNA sequencing analysis was performed. This showed the wild-type transcript coexisting with an alternative out-of-frame transcript, that is subjected to nonsense-mediated-decay. Thus, the *HNRNPK* variant could be interpreted as p.Tyr72fs*19 and can be classified as likely pathogenic.

### 4.3 Clinical neuropsychological assessment

Although the patient was easily distracted, sometimes staring briefly or looking as if something or someone was present in the corner of the room, she had sufficient motivation and attention to complete the procedures. **Supplementary File 2** shows the results of the clinical neuropsychological assessment. In summary, overall intellectual functioning was at a moderate level, with corresponding levels of adaptive and social functioning. There was slow information processing speed, restricted verbal expression, and limited comprehension, and a very low level of executive functioning. Registration of sensory information was severely hampered by problems with posture, movement, and balance; associated with low endurance and weak muscle tone. There was also difficulty in self-modulating the sensory information to be processed. As a result, behavior resulted directly from all sensory input much more than in normative controls. Trauma and other stressors were ruled out.

### 4.4 Therapeutic interventions

At the age of 40, the patient and her parents moved from a big city to a quiet rural area. Home care assistance was initiated and the patient was well reintegrated into day care. At the age of 41, the ID physician, clinical neuropsychologists, and psychiatrist jointly concluded that the symptoms could be stress-related (‘reactive psychosis’), triggered by former independent living without sufficient guidance for her intellectual disability. As treatment with pipamperone had shown insufficient effect, it was changed to prophylactic aripiprazole (20 mg/day). After neuropsychological assessments, the potential for further recovery was sought in the patient’s expanded engagement in meaningful, practical actions at home and in daytime activities, offered at a slow speed and repeated frequently (ingraining skills). In addition, individualized psychomotor therapy in combination with sensory integration therapy was started, to explicitly promote (registration of stimuli from) posture, movement and muscle tone. Movement was also considered to be generally beneficial for body weight control and to improve endurance (motor function), both to release tension and to stimulate the adequate level of engagement and arousal. At present (age 45), no behavioral decline is reported anymore.

## 5 Discussion

We described the clinical course of a woman with the ultrarare AUKS. To our knowledge, this is the oldest reported case of AUKS to date. The novel *HNRNPK* variant identified in this patient was classified as likely pathogenic. This intronic variant affects the splice acceptor site, resulting in an alternative out-of-frame transcript and consequent haploinsufficiency. Au–Kline syndrome is caused by haploinsufficiency of *HNRNPK*, most commonly due to loss-of-function variants, which are typically associated with a characteristic phenotype featured by intellectual disability, distinctive facial features, and multisystem involvement. In contrast, missense variants have been associated with milder or atypical presentations. The somatic characteristics of the patient were consistent with features of typical AUKS (see also **Supplementary File 3**) and the severity of intellectual disability was more pronounced compared to individuals harboring missense variants. Therefore, the novel *HNRNPK* variant was causative for AUKS.

In addition to this genotype–phenotype correlation, the cognitive and behavioral characteristics provide new information. Based on extensive clinical neuropsychological assessment, features of information processing, particularly sensory and motor functioning, were identified in this individual with mild to severe intellectual disability. It was useful to analyze the behavioral symptoms in terms of cognitive information processing and underlying genetic conditions, rather than interpreting them as a psychopathological condition in itself. In addition, (premorbid) stereotypic behaviors (leg and hand movements; facial expressions) were identified and considered likely to reflect basic information processing needs (stimulus seeking or modulation, distraction, symptoms of stress or arousal). Individualized treatment was then initiated to stimulate sensory information processing and improve motor skills. The patient’s home care and daily activities were further adapted to her profile of (sensory, motor, adaptive, cognitive) information processing. Between the ages of 41 and 45, there was a gradual recovery from symptoms, and her mood remained cheerful. The patient is currently (age 45) functioning at almost the same level as before the deterioration (at age 32), and no behavioral decline is reported anymore.

A major strength of this case report is the diagnostic approach, combining trio-based genetic testing with comprehensive clinical neuropsychological assessment in adulthood, in addition to primary and psychiatric care. The literature on AUKS has primarily focused on somatic and medical characteristics, while cognitive and behavioral functioning has largely been described at an observational level only (e.g., hypotonia, developmental delay) and almost exclusively in childhood (1, 2, 6). The present case extends the phenotypic spectrum of *HNRNPK*-related disorders into mid-adulthood and provides an in-depth characterization of functioning using formal neuropsychological methods. Furthermore, behavioral deterioration was interpreted from a developmental and information-processing perspective. This aligns with broader research on adults with intellectual disability, in which behavioral regression is conceptualized as a result of the interaction between neurodevelopmental vulnerability and contextual overload, rather than primary psychiatric pathology or neurodegeneration (7–9). The applied diagnostic approach proved clinically meaningful and facilitated targeted, individualized interventions.

Several limitations should be acknowledged. As a single-case report, generalizability is inherently limited. Although our findings are consistent with models of stress-related regression in adults with developmental disorders (7, 8), it remains to be seen to what extent the observed neuropsychological profile and therapeutic response are representative of other individuals with AUKS, particularly given the associated genotypic and phenotypic variability (1–3, 6). In addition, although improvement followed both pharmacological changes and non-pharmacological interventions, the relative contribution of each component cannot be disentangled retrospectively. In future case reports, evaluations would ideally include simultaneous and longitudinal medical and psychiatric assessments (including systematic review of medication use, potential side effects, and staged withdrawal), structured behavioral observations (e.g., antecedent–behavior–consequence [ABC] analyses, behavior rating scales), as well as repeated formal assessments of information processing and functioning. Nevertheless, similar to reports in other neurodevelopmental disorders, reductions in environmental stress and the enhancement of psychomotor regulation may have been more decisive than antipsychotic treatment alone (7, 9). Additional adult cases and longer follow-up may help to further substantiate whether later-life risks occur in this ultrarare condition, as has been suggested for some - but not all - genetic developmental disorders (10).

In conclusion, this case highlights that the genetic and neuropsychological identification of a neurodevelopmental disorder, together with tailored psychomotor and contextual interventions, can substantially improve psychiatric management and daily functioning, and mitigate behavioral decline in adulthood, even following a prolonged diagnostic delay.

## 6 Patient perspective

Given the individual’s intellectual capacities, direct verbal self-report regarding her treatment was not feasible. Her parents, who also serve as her caregivers and legal representatives, provided frequent written correspondence offering their perspective on the treatment. “At the age of 43, our daughter is very stable, both physically and emotionally. She hardly talks ‘into the void’ anymore, except occasionally at the end of the day when she is processing the stimuli of the day. Her communication skills have improved markedly. Moreover, the quality she was always known for and loved for—her warm interest in other people—has fully returned. Another positive development, is that she no longer dreads changes; in the past, she found this extremely difficult.”

## Data Availability

The authors declare that all data that can reasonably be shared are provided in this article and its **Supplementary Files (1, 2, and 3)**. Additional data supporting the findings of this study are available from the corresponding author upon reasonable request.
