# Supplementary material for: Case Report: A novel HNRNPK gene variant causing Au-Kline syndrome; the genotype and phenotype of the oldest individual ever described
Source: Front Psychiatry. 2026 Aug 7;17:1864501. doi: 10.3389/fpsyt.2026.1864501 (PMC13494470; doi:10.3389/fpsyt.2026.1864501)
Supplement: Supplementary file 1 [file Supplementaryfile1.docx]

**Supplementary Material 1. Materials and methods genetic testing**

Following routine diagnostic procedures as described by Neveling et al. (2013). Essentially, DNA was sequenced on an Illumina system, after exome enrichment with the Agilent SureSelectXT All Exon 50Mb kit V5. Reads were aligned to the Hg19 reference genome with BWA and variants were called using GATK and annotated using an in-house developed pipeline.

WES analysis statistics:

Total number of variants:	124749

Genepanel version:	DG2.13 (Intellectual Disability)

Variants in genepanel:	8628

Low frequency variants (<1% ExAC):	5591

Low Frequency and exonic (+/-10bp)	135

Low frequency and exonic and DE NOVO:	1

Reference

Neveling K, Feenstra I, Gilissen C, et al. A post-hoc comparison of the utility of sanger sequencing and exome sequencing for the diagnosis of heterogeneous diseases. Hum Mutat. 2013;34(12):1721-1726. doi:10.1002/humu.22450
