# Supplementary material for: Case Report: A novel HNRNPK gene variant causing Au-Kline syndrome; the genotype and phenotype of the oldest individual ever described
Source: Front Psychiatry. 2026 Aug 7;17:1864501. doi: 10.3389/fpsyt.2026.1864501 (PMC13494470; doi:10.3389/fpsyt.2026.1864501)
Supplement: Supplementary file 2 [file Supplementaryfile2.docx]

**Supplementary Material 2. Neuropsychological test results of the patient**

| **Domain** | **Test** | **Reference** | | **Domain of  measurement** | **Score label** |  | |
| --- | --- | --- | --- | --- | --- | --- | --- |
|  |  |  | |  |  | **Standardized score^a^** | |
| Intelligence | WAIS-IV-NL | Wechsler (2012) | | FSIQ | Moderate intellectual disability ^b^ | 42 – 52 | |
|  |  |  | | VCI | Moderate to mild intellectual disability | 45 – 60 | |
|  |  |  | | PRI | Moderate to mild intellectual disability | 41 – 56 | |
|  |  |  | | WMI | Moderate to mild intellectual disability | 42 – 61 | |
|  |  |  | | PSI | Moderate intellectual disability | 42 – 52 | |
|  |  | |  |  |  | **Raw score** | **Standardized score** |
| Adaptive functioning | Vineland-II Adaptive Behavior Scales, semi-structured interview | | Sparrow et al. (2008) | Communication   - Receptive - Expressive - Written   Daily living skills   - Personal - Domestic - Community   Socialization   - Interpersonal Relationships - Play and Leisure Time - Coping Skills   Motor skills   - Fine motor skills - Gross motor skills   Overall Adaptive behavior | Exceptionally low score **^c^**  Exceptionally low score  Exceptionally low score  Exceptionally low score  Exceptionally low score | 76  182  16  186  62  89  120  58  113  100  126 | 38  40  42  50  35 |
|  |  | |  |  |  | **Raw score** | **Z-scores** |
| Sensory processing | SP-NL^d^ | | Brown & Dunn (2013); Rietman (2013) | *Quadrants*   - Lack of registration - Sensory seeking - Sensory sensitivity - Sensation avoiding   *Sensory Processing (input)*   - Auditory Processing - Visual Processing - Vestibular Processing - Touch Processing - Multisensory Processing - Oral sensory Processing   *Modulation (throughput)*   - Sensory Processing Related to Endurance/Tone - Modulation Related to Body Position & Movement - Modulation of Movement Affecting Activity Level - Modulation of Sensory Input Affecting Emotional Responses - Modulation of Visual Input Affecting Emotion Responses and Activity Level   *Behavior and Emotional Responses (output)*   - Emotional/Social Responses - Behavioral Outcomes of Sensory Processing - Items Indicating Thresholds for Response | Much more than others ^e^  Comparable with others  More than others  More than others  Comparable with others  More than others  Much more than others  More than others  More than others  Comparable with others  Much more than others  Much more than others  More than others  Comparable with others  More than others  Comparable with others  Much more than others  Comparable with others | 41 / 75  123 / 130  77 / 100  105 / 145  34 / 40  32 / 45  40 / 55  74 / 90  25 / 35  59 / 60  23 / 45  31 / 50  19 / 35  17 / 20  14 / 20  77 / 85  14 / 30  15 / 15 | ≥2*SD*  ≤1*SD*  1-2*SD*  1-2*SD*  ≤1*SD*  1-2*SD*  ≥2*SD*  1-2*SD*  1-2*SD*  ≤1*SD*  ≥2*SD*  ≥2*SD*  1-2*SD*  ≤1*SD*  1-2*SD*  ≤1*SD*  ≥2*SD*  ≤1*SD* |
| Memory | LLT ^f^ | | Kessels et al. (2012) | Visual spatial learning and memory | NA | NA | |
|  |  | |  |  |  | **Raw score** | **Percentile** |
| Visual constructive | Beery Visual-Motor Integration (VMI) | | Beery & Beery (2010) | Visual-Motor Integration  Visual Perception  Motor Coordination | Exceptionally low score^c^  Exceptionally low score  Exceptionally low score | 10  13  10 | <0.1  <0.1  <0.1 |
|  |  | |  |  |  | **Raw score** | **T-score** |
| Executive functioning | BRIEF-A^e^ | | Scholte & Noens (2011) | - Inhibition - Cognitive flexibility - Emotion regulation - Self-evaluation   **Behavior regulation index**   - Taking initiative - Working memory - Planning & Organizing - Task evaluation - Tidiness   ***Metacognitive index***  ***Total Score*** | Non-deviant score ^g^  Non-deviant score  Non-deviant score  Non-deviant score  **Non-deviant score**  Deviant score  Deviant score  Deviant score  Deviant score  Non-deviant score  **Deviant score**  **Non-deviant score** | 13  13 10  10  ***46***  19  22  22  15  16  ***94***  ***140*** | 57  62 40  54  ***51***  71  81  67  72  58  ***72***  ***64*** |
|  | BRIEF-A ^j^ | | Scholte & Noens (2011) | - Inhibition - Cognitive flexibility - Emotion regulation - Self-evaluation   **Behavior regulation index**   - Taking initiative - Working memory - Planning & Organizing - Task evaluation - Tidiness   ***Metacognitive index***   - ***Total Score*** | Deviant score ^g^  Non-deviant score  Non-deviant score  Non-deviant score  **Non-deviant score**  Non-deviant score  Deviant score  Deviant score  Deviant score  Non-deviant score  **Deviant score**  **Non-deviant score** | 16  10  11  11  **48**  14  22  22  15  14  **87**  **135** | 67  52  42  57  **53**  56  81  67  72  53  **67**  **63** |
|  | ABCL-A ^e^ | | Achenbach & Rescorla (2003) | Anxious/ depressed  Withdrawn/depressed  Somatic complaints  Thought problems  Attention problems  Aggressive behavior  Rule-breaking behavior  Social problems  Internalizing  Externalizing  ***Total*** | Non-deviant score ^h^  Non-deviant score  Non-deviant score  Deviant score  Deviant score  Non-deviant score  Non-deviant score  Non-deviant score  Non-deviant score  Non-deviant score  ***Increased score*** | 0  1  3  8  21  3  2  0  4  5  ***50*** | 48  53  ***60*** |
|  | ABCL-A^h^ | | Achenbach & Rescorla (2003) | Anxious/ depressed  Withdrawn/depressed  Somatic complaints  Thought problems  Attention problems  Aggressive behavior  Rule-breaking behavior  Social problems  Internalizing  Externalizing  ***Total*** | Non-deviant score ^h^  Non-deviant score  Non-deviant score  Deviant score  Deviant score  Non-deviant score  Increased score  Non-deviant score  Non-deviant score  Non-deviant score  ***Increased score*** | 4  5  0  9  20  3  6  1  9  10  ***58*** | 55  58  ***62*** |
|  |  | |  |  |  | **Raw score** | **POP-plus score ^j^** |
| Social competence | SRZ-P | | Kraijer & Kema (1994); Kraijer & Kema (2004) | Self-Sufficiency I (primary)  Self-Sufficiency II (secondary)  Verbal numeric  Total Score | Moderate intellectual disability score ^k^  Moderate intellectual disability score  Moderate intellectual disability score  Moderate intellectual disability score | 5  1  3  13 | 5  4  5  5 |
|  |  | |  |  |  | **Raw score ^l^** | |
| Psychopathology and personality | PIMRA ^d^ | | Matson et al. (1994) | Schizophrenia  Affective disorder  Psychosexual disorder  Adaptation disorder  Anxiety disorder  Somatoform disorder  Personality disorder  Inadequate adjustment  ***Total*** |  | 3 / 7  1 / 7  0 / 7  1 / 7  1 / 7  0 / 7  1 / 7  1 / 7  ***8 / 56*** | |

*Notes.*

^a^ Wechsler intelligence quotients*; M* = 100, *SD* = 15.

^b^ ICD-11 classification of intellectual disability (Girimaji & Pradeep, 2018)

^c^ Score labels according to Guilmette et al. (2020)

^d^ Proxy, parent report

^e^ Score labels according to SP-NL (Rietman, 2013); 1-2*SD* = more than others; ≥2*SD* = much more than others

^f^ Canceled due to insufficient understanding of test instructions

^g^ Classifications *T*≤65 is non-deviant; *T*>65 deviant, according to Scholte & Noens (2011)

^h^ Classifications *T*≥ 64 is deviant (clinical range); 60≤*T*<64 is increased; *T*<60 is non-deviant, according to Achenbach & Rescorla (2003)

^i^ Proxy, counselors report

^j^ Standard POP-Plus scores, from 12 years and older, according to Kraijer & Kema (2004)

^k^ Based on the empirical school grade distribution (*M* = 6.33, *SD* = 1.33)

^l^ A psychopathological level is indicated by the presence of at least four of the seven symptoms on the subscale (Matson et al., 1994)

*Abbreviations.* WAIS-IV-NL, Wechsler Intelligence Scale, fifth edition, Dutch version (Wechsler, 2012); FSIQ, Full Size Intelligence Quotient; VCI, Verbal Comprehension Index; PRI, Perceptual Reasoning Index; WMI, Working Memory Index; PSI, Processing Speed Index; SP-NL, Sensory Profile, Dutch version; *SD,* Standard Deviation; LLT, Location Learning Test; NA, Not Applicable; Beery VMI, Beery Visual Motor Integration; BRIEF-A, Behavior Rating Inventory of Executive Function, Adult version; ABCL, Adult Behavior Checklist; SRZ-P, Social Competence Rating Scale for persons with an intellectual disability, Proxy report; PIMRA, Psychopathology Inventory for Mentally Retarded Adults; *M,* Mean.

**References**

Achenbach, T. M., & Rescorla, L. A. (2003). *Manual for the ASEBA Adult Forms & Profiles.* University of Vermont, Research Center for Children, Youth, & Families.

Beery, K. E. & Beery, N. A. (2010). *Beery VMI, Administration, Scoring, and Teaching Manual (6th Edition).* Pearson Clinical Assessment.

Brown, C. E., & Dunn, W. (2013) *SP-NL. Sensory profile, herziene Nederlandse editie*. Handleiding. Pearson.

Girimaji, S. C., & Pradeep, A. J. (2018). Intellectual Disability in International Classification of Diseases-11: A Developmental Perspective. *Indian Journal of Social Psychiatry*

*34*(Suppl 1), S68-S74.

Guilmette, T. J., Sweet, J. J., Hebben, N., Koltai, D., Mahone, E. M., Spiegler, B. J., Stucky, K., Westerveld, M. & Conference Participants. (2020). American academy of clinical

neuropsychology consensus conference statement on uniform labeling of performance test scores. *The Clinical Neuropsychologist, 34*(3), 437-453. <https://doi.org/10.1080/13854046.2020.1722244>

Hendriks, M., Kessels, R., Gorissen, M. & Schmand, B. (2014). *Neuropsychologische diagnostiek: De klinische praktijk*. Boom.

Kessels, R. P. C., Bucks, R. S., Willison, J. R., & Byrne, L. M. T. (2012). *Location Learning Test Herziene Uitgave – Handleiding*. Hogrefe Uitgevers B.V.

Kraijer, D. W., & Kema, G. N. (1994). *Sociale redzaamheidsschaal voor zwakzinnigen SRZ.* Swets & Zeitlinger.

Kraijer, D. W., Kema, G. N., & de Bildt, A. A. (2004). *SRZ, SRZ-i, Sociale redzaamheidsschalen Handleiding*. Pearson Assessment and Information B.V.

Kraijer, D. W., & Kema, G. N. (2004). *Sociale Redzaamheidsschaal-Z voor hoger niveau. SRZ-P Handleiding*. Vierde uitgebreide uitgave. Pearson Assessment and Information B.V

Matson, J., van Minnen, A., & Hoogduin, K. (1994). Handleiding: De Nederlandse versie van de Psychopathology Inventory for Mentally Retarded Adults. IDC

Rietman, A. (2013*). Sensory Profile, herziene Nederlandse editie.* Pearson Assessment and Information B.V.

Scholte, E., & Noens, I. (2011). *BRIEF-A – Vragenlijst executieve functies voor volwassenen. Handleiding*. Hogrefe Uitgevers B.V.

Sparrow, S.S., Cichetti, D. V., & Balla, D. A. (2008). *Vineland TM-II Vineland Adaptive Behavior Scales (second edition)*, PsychCorp, Pearson Clinical Assessment [USA normen]

Wechsler, D. (2012). *WAIS-IV Nederlandse Bewerking – Afname en Scoringshandleiding*. Pearson Assessment and Information B.V.

Wechsler D. (2012). *WAIS-IV Nederlandse Bewerking – Technische Handleiding*. Pearson Assessment and Information B.V.
