# Supplementary material for: Case Report: A novel HNRNPK gene variant causing Au-Kline syndrome; the genotype and phenotype of the oldest individual ever described
Source: Front Psychiatry. 2026 Aug 7;17:1864501. doi: 10.3389/fpsyt.2026.1864501 (PMC13494470; doi:10.3389/fpsyt.2026.1864501)
Supplement: Supplementary file 3 [file Supplementaryfile4.doc]

Article ID 1864501
Type Setters Request: B (29.07.2026)

**References that are cited exclusively in Supplementary File 3**

11. Pua HH, Krishnamurthi S, Farrell J, Margeta M, Ursell PC, Powers M, et al. Novel interstitial 2.6 Mb deletion on 9q21 associated with multiple congenital anomalies. *Am J Med Genet A.* (2014) 164A:237-42. doi: 10.1002/ajmg.a.36230

12. Hancarova M, Puchmajerova A, Drabova J, Karaskova E, Vlckova M, Sedlacek Z. Deletions of 9q21.3 including NTRK2 are associated with severe phenotype. *Am J Med Genet A*. (2015) 167A:264-7. doi:10.1002/ajmg.a.36797

13. Lange L, Pagnamenta AT, Lise S, et al. A de novo frameshift in HNRNPK causing a Kabuki-like syndrome with nodular heterotopia. *Clin Genet*. (2016) 90:258-62. doi:10.1111/cge.12773

14. Miyake N, Inaba M, Mizuno S, Shiina M, Imagawa E, Miyatake S, et al. A case of atypical Kabuki syndrome arising from a novel missense variant in HNRNPK. *Clin Genet.* (2017) 92: 554-5. <https://doi.org/10.1111/cge.13023>

15. Dentici ML, Barresi S, Niceta M, Pantaleoni F, Pizzi S, Dallapiccola B, et al. Clinical spectrum of Kabuki-like syndrome caused by *HNRNPK* haploinsufficiency. *Clin Genet.* (2018) 93:401–7. <https://doi.org/10.1111/cge.13029>

16. Shashi V, Schoch K, Spillmann R, Cope H, Tan QK, Walley N, et al. A comprehensive iterative approach is highly effective in diagnosing individuals who are exome negative. *Genet Med*. (2019) 21:161-72. doi:10.1038/s41436-018-0044-2

17. Yamada M, Shiraishi Y, Uehara T, Suzuki H, Takenouchi T, Abe-Hatano C, et al. Diagnostic utility of integrated analysis of exome and transcriptome: Successful diagnosis of Au-Kline syndrome in a patient with submucous cleft palate, scaphocephaly, and intellectual disabilities. *Mol Genet Genomic Med*. (2020) 8:e1364. doi:10.1002/mgg3.1364

18. Järvelä I, Määttä T, Acharya A, Leppälä J, Jhangiani SN, Arvio M, et al. Exome sequencing reveals predominantly de novo variants in disorders with intellectual disability (ID) in the founder population of Finland. *Hum Genet* (2021) 140:1011–29. <https://doi.org/10.1007/s00439-021-02268-1>

19. Wallerstein R, Shih LY, Fong MH, Zheng S, Poon E. A new case of Okamoto syndrome. *Clin Dysmorphol.* (2005) 14(2):85-7. doi:10.1097/00019605-200504000-00006

20. Wallerstein R, Rhoads F. Natural history of a child with Okamoto syndrome. *Clin Dysmorphol.* (2013) 22:127-8. doi:10.1097/MCD.0b013e32836127d0

21. Conteh FS, Tyagi R, Brooks SS. A new finding of a tethered cord in a patient with Okamoto syndrome. *Clin Dysmorphol*. (2017) 26:110-3. doi:10.1097/MCD.0000000000000147

22. Markouri M, Karpathios T, Dinopoulos A, Attilakos A, Fretzayas A, Bakoula C, et al. Okamoto syndrome in a girl of Caucasian origin. *Dev Med Child Neurol.* (2018) 50:950-2. [doi.org/10.1111/j.1469-8749.2008.03147.x](https://doi.org/10.1111/j.1469-8749.2008.03147.x)

23. Taylor J, Aftimos S. A newborn with likely Okamoto syndrome. *Clin Dysmorphol*. (2010) 19:103-6. doi:10.1097/MCD.0b013e328337bb8d

24. Okamoto N. Okamoto syndrome has features overlapping with Au–Kline syndrome and is caused by HNRNPK mutation *Am J Of Med Genet A*. (2019) 179:822-6. doi:10.1002/ajmg.a.61079

25. Maystadt I, Deprez M, Moortgat S, Benoît V, Karadurmus D. A second case of Okamoto syndrome caused by HNRNPK mutation. *Am J Med Genet A*. (2020) 182:1537-9. doi:10.1002/ajmg.a.61568
